# A Post-Fellowship Support Framework for Rural Doctors: the Queensland experience

**DOI:** 10.12688/mep.20025.2

**Published:** 2024-11-20

**Authors:** Dilip Dhupelia, Ansmarie Van Erp, James Collins, Tarun Sen Gupta

**Affiliations:** 1 Darling Downs Health, Queensland Country Practice, Brisbane, QLD, 4001, Australia; 2 College of Medicine & Dentistry, James Cook University, DOUGLAS, Queensland, 4811, Australia

**Keywords:** Rural medicine, retention, post-Fellowship, workforce, support

## Abstract

**Background:**

International workforce shortages have prompted many initiatives to recruit, train and retain rural doctors, including Australia’s emerging National Rural Generalist Pathway. This project explored an important component of retention, rural doctors' post-Fellowship support needs, to develop and validate a post-Fellowship support framework. There has been considerable international attention on social accountability in medical education and how medical schools and other institutions can address the needs of the communities they serve. The recognition that rural and remote communities globally are underserved has prompted numerous educational approaches including rurally focused recruitment, selection, and training. Less attention has been paid to the support needs of rural doctors and how they can be retained in rural practice once recruited.

**Methods:**

The project team reviewed international and Australian rural workforce and medical education literature and relevant policy documents to develop a set of guiding principles for a post-Fellowship support framework. The project utilised a mixed methods approach involving quantitative and qualitative methodologies; this paper focuses on the qualitative aspects. A range of rural doctors, administrators, and clinicians, working in primary and secondary care, across multiple rural locations in Queensland were invited to participate in interviews. Thematic analysis was undertaken.

**Results:**

The interviews validated ten interconnected guiding principles which enabled development of a grounded, contextually relevant approach to post-Fellowship support. This framework provides a blueprint for a retention strategy aiming to build a strong, skilled, and sustainable medical workforce capable of meeting community needs. Four themes emerged from the inductive thematic analysis: connecting primary and secondary care; valuing a rural career; supporting training and education; and valuing rural general practice.

**Conclusions:**

The ten principles were designed in the real-world context of a mature Queensland Rural Generalist Pathway. The four themes will facilitate engagement and consultation with rural stakeholders to develop appropriate retention and support strategies.

## Introduction

Virtually every country worldwide is experiencing shortages of rural doctors and other health providers with consequent effects on service delivery and community health outcomes. The recognition of rural generalist doctors skilled in primary care, public health and advanced specialised care is one approach to addressing this global deficit.

In 2007, Queensland was the first Australian state to establish a Rural Generalist Pathway - an effective, sustainable training pathway leading to a vocationally registered career in Rural Generalist Medicine ^1^. A Rural Generalist needs to be competent in a scope of practice able to meet the needs of their community which typically will have limited access to services available in larger centres and be influenced by geographic and demographic factors. The scope of practice is broad, providing medical services capable of responding to the health needs of an entire community, i.e., a ‘generalist’ scope of practice which integrates primary and secondary care ^2^. The Queensland pathway is well-developed having produced a critical mass of 234 graduates who have achieved vocational recognition via College Fellowship. There are currently over 384 trainees, many filling critical vacancies in rural hospitals, i.e. providing a significant component of the clinical service whilst undertaking supervised training ^3^.

In Queensland, the state government funds hospitals and primary care clinics in many rural locations while the Commonwealth supports private General Practitioners (GPs) through Medicare funding. Rural communities may have one or both models, with many Queensland rural doctors working in both sectors ^3^.

### National Rural Generalist Pathway

Australia has a strong commitment to a national framework for rural generalism, the National Rural Generalist Pathway (NRGP), which will build a skilled workforce like the established program in Queensland ^4^. While there are clear demographic differences between ‘rural’ and ‘remote’ communities the national pathway does not make this distinction, so the term ‘rural’ will be used throughout this paper.

Australia has two General Practice colleges, the Australian College of Rural and Remote Medicine (ACRRM) and the Royal Australian College of General Practitioners (RACGP), who collectively define a ‘Rural Generalist’ as:

“a medical practitioner who is trained to meet the specific current and future health care needs of Australian rural and remote communities, in a sustainable and cost-effective way, by providing both comprehensive general practice and emergency care and required components of other medical specialist care in hospital and community settings as part of a rural health care team.”

The definition, the so-called Collingrove Agreement, enables a pathway supporting doctors to practise and live rurally and ensures they are supported to attain and maintain the advanced skills required to meet community need ^4, 5^.

In Australia, as in many other countries, GPs and rural doctors generally undertake formal postgraduate training and complete relevant summative assessments. In Australia, this training is a minimum 3 years for general practice and 4 years for rural medicine, and auspiced by the relevant Colleges who also administer barrier examinations. Completion of training and assessment confers Fellowship of the College and an ability to undertake independent, unsupervised, vocationally recognized practice.

### Attracting and retaining rural doctors

Many workforce strategies focus on recruitment and retention via education and training. The high turnover of rural health professionals exacerbates workforce shortages, highlighting the critical importance of retention strategies ^6^. A recent scoping review noted many more papers on recruitment compared with retention, observing that effective strategies were feasible in a range of settings but dependent on a national commitment to act ^7^. These authors described the importance of the social accountability of medical schools in facilitating a sustainable rural and remote workforce. They cite the WHO definition of social accountability - ‘the obligation to direct their education, research and service activities towards addressing the priority health concerns of the community, the region or the nation they have a mandate to serve’ - noting evidence to support rural focused recruitment and rural training including rural experiences.

 A 2021 systematic review of 34 papers, all observational studies, concluded that policy makers would be ‘prudent to strengthen rural training pathways and limit the use of strongly coercive interventions’ ^8^. This review found evidence that educational interventions including preferential selection of rural health professional students and distributed training in rural sites were associated with increased rural retention, as well as recruitment. They did note that ‘strongly coercive interventions are associated with comparatively lower rural retention than interventions that involve less coercion.’

A review of retention of GPs in remote Canada and Australia identified six synthesised findings: peer and professional support; organizational support; uniqueness of remote lifestyle and work; burnout and time off; personal family issues; and cultural and gender issues ^9^. These authors observed their findings spanned a spectrum of policy domains and service responsibilities, arguing that ‘a central coordinating body could well be placed to implement a multi factorial retention strategy.’ Their study identified excess bureaucracy and lack of organisational support and resourcing as the key reasons for doctors leaving remote practice, with burnout, cultural and gender issues, and family issues also having an impact on retention.

Veitch and Battye noted in 2008 that, ‘Retention is the successful outcome of recruitment when health professionals are recruited into rural practice and remain for extended periods.’ They cite numerous studies which recognise that retention involves a different set of issues from recruitment, observing that ‘decisions to take up rural practice (recruitment) are made outside of the contextual setting of rural practice, whereas decisions to remain (retention) occur within that setting and are based on experience there’ ^10^.

The same authors describe retention decisions as ‘a dynamic equilibrium of positive and negative factors,’ noting that the equilibrium can be upset by changes in workload and role changes. They further set out 26 factors that influence retention, grouped into the broad dimensions of security, freedom, and identity ^10^.

## Background

This project explored an important component of retention, the post-Fellowship support needs of vocationally registered Queensland rural doctors. Two key workforce bodies, Queensland Country Practice (Queensland’s Rural Generalist Coordination Unit), and Health Workforce Queensland (the state rural workforce agency), pooled their collective expertise in rural workforce using a Delphi method to define a set of Guiding Principles, tailored to the rural Queensland context ( Table 1). Further details of the Delphi method are in the detailed project report ^11^.

**Table 1.** Guiding Principles for a Post-Fellowship Support Framework.

Principle 1	Collaboration and coordination between primary and secondary care is vital
Principle 2	Rurally based career paths are recognised and valued
Principle 3	Job satisfaction is a critical element in working in rural and remote areas
Principle 4	Support is provided to attain advanced skills in order to meet community need.
Principle 5	The ability to attain, maintain and upgrade procedural skills in hospitals or general practices is supported to meet community needs
Principle 6	The ability to work across hospital and general practice facilities to provide coordinated care is supported
Principle 7	Education and training needs are supported
Principle 8	Professional peer support is available
Principle 9	The viability of existing rural general practice is enhanced
Principle 10	Clinical leadership capability is recognised, enhanced, encouraged, and supported

These guiding principles were used to develop a post-Fellowship support framework for Queensland rural doctors. This framework provides a blueprint for a retention strategy aiming to build a strong, sustainable, and skilled medical workforce capable of meeting community needs.

This project aimed to validate and evaluate the interconnected guiding principles. Post–Fellowship rural doctors and registrars were asked to comment on the importance of each principle and to outline any further principles necessary to support rural generalists. These findings were used to inform the educational approach, development, and implementation of a bespoke support framework relevant to current and future rural generalists in both the private and public sectors.

## Methods

A project team was established with representatives from the partner organizations which had statewide roles in rural health workforce, policy, and training. Members of the team had backgrounds in health workforce, medical administration, and research / project management, and had professional connections with a number of participants in the project. These participants in turn would have had knowledge of the partner organizations and members of the project team and their roles.

Informed consent was provided by all participants. Verbal consent was sought (and audio-recorded) from those who participated in the interviews. Confidentiality was discussed, with an explanation that responses would be de-identified as appropriate. Participants who completed the online survey questions provided written consent.

This project aimed to validate the guiding principles with Queensland stakeholders in the context of a mature Queensland Rural Generalist Pathway and an evolving National pathway. Interviews and an on-line survey ( Qualtrics™) were used to develop themes which reflect the key areas of support that rural doctors identify as necessary to assist post-Fellowship doctors to practise in accordance with the Collingrove Agreement ^5^. These themes will form the foundations used to design the post-Fellowship support framework.

A purposive sampling strategy was used with a variety of rural doctors invited to contribute in order to maximise the likelihood of contributions reflecting the diversity of rural doctors working across primary care, secondary care, and Aboriginal Medical Services (AMS). Participants included administrators (Directors of Medical Services) and clinicians (Rural Generalists and GPs).

The pilot project utilised a mixed methods approach using qualitative and quantitative methods.

The qualitative component, which is the focus of this paper, involved in-depth one-on-one semi-structured interviews by a doctor from the project team. An interview guide (see *Extended data ^12^*) was developed to:

- Document career history.
- Ascribe a weighting for each of the proposed guiding principles used in developing a post-Fellowship support framework; and
- Discuss awareness and understanding of the National Rural Generalist Pathway and the Collingrove definition.

The questions were piloted with two experienced rural doctors.

A variety of rural locations were deliberately chosen for the project interviews: so-called ‘Town A’ (a single site) and ‘Town B’ (an amalgamation of six separate sites across Queensland). Town A was an exemplar site with recognised success in delivering innovative local services, excellent leadership, and integration of rural medical workforce across public and private sectors, hospital, and primary care. Various employment models were utilised resulting in high levels of retention and job satisfaction.

Town B was a ‘virtual town’ consisting of six locations with similar demographics to Town A, but with varying degrees of clinical leadership, staff stability, job satisfaction, and collaboration across sectors. Integrated models of care were less well developed with a relatively smaller number of doctors working across both primary and secondary care. The Town B sites were chosen to explore local inhibitory factors and to ensure geographic representation across Queensland. ( Table 2)

**Table 2.** Selection criteria for sites participating in the qualitative component of the pilot.

**Selection Criteria for Town A** Town A was an actual town in a rural location in Queensland comprising of a single site that met the following selection criteria: • an MMM 4 -7 location • the existence of at least one private general practice and a public hospital • established clinical leadership • existence of well-established connection, collaboration, and good relationship between primary and secondary care • a general practice with a history of involvement in training through mentoring medical students, junior doctors, and GP registrars • doctors working across both hospital and general practice settings **Selection Criteria for Town B sites** ‘Town B’ was comprised of six different rural towns in Queensland that met the following selection criteria: *1.* A mixed sample of geographical location and remoteness including aspects such as: • a range of MMM 4-7 locations i.e., rural (non-metropolitan, with population less than 15 000) or remote • sites from 6 different Hospital and Health Services • includes an Aboriginal Medical Service • reflect a mixed sample of the following employment models: • predominantly Senior Medical Officer (SMO) workforce model • Medical Superintendent / Medical Officer Private Practice (MS/MOPP) workforce model • the existence of at least one private general practice and a public hospital • GP Registrar workforce • Rural Generalist workforce (i.e., doctors whose practice meets the Queensland recognised discipline of Rural Generalist medicine) • Towns with heavy reliance on locums *2.* A mixed sample of towns with differing relationships betw those with: • a well-established connection • no connection • a historically good relationship that has now broken down or vice versa

The choice of Town A and Town B reflected a purposive sampling strategy to get the best representation of communities across Queensland in order to ensure the study encompassed both high-functioning communities and other types of communities. 

The Town A and Town B sites were selected on the basis of the project team’s knowledge of local workforce supported by a desktop audit. A community profile developed for each site, included geographic factors, population demographics, Socio-Economic Indexes for Areas (SEIFA) ^13^ and a description of the current health workforce and services.

### Participants

Participants were recruited by members of the project team in mid-2020 using the selection criteria described above ( Table 2). Contact details (email addresses and telephone numbers) were sourced from databases held by the project team.

A total of 31 doctors (15 female and 16 male) from Towns A and B were interviewed. One additional doctor who had confirmed their participation later withdrew. In Town A, all clinically active doctors participated in the interview process. Three doctors (a private GP, a rural generalist, and a Director of Medical Services / medical administrator) from each of the six Town B sites were recruited. In addition, a GP working solely in an Aboriginal Medical Service was also interviewed.

### Approach

Semi-structured interviews were conducted between August and October 2020 by two doctors (DD, JC – both male and with backgrounds in general practice, medical administration, and health workforce) from the project team. Interviews were conducted face-to-face in person in Town A and via either telephone or videoconference using Microsoft Teams for Town B sites. Interviews generally lasted 60–90 minutes and were transcribed verbatim. The interviewers recognised that similar responses began to appear consistently across interviews which indicated that the data saturation was being achieved and no further exploration was warranted. Recordings were made of each interview which were used to check transcriptions for accuracy. Participants were invited to a face-to-face presentation or teleconference to discuss the early data analyses and provide comments or corrections.

Interviewees were asked to describe their rural medical experience. Those with at least ten years’ rural experience were asked to further comment on any changes to rural medical practice over this time.

The interviews went on to explore the draft guiding principles. Prompting questions were used to explore interviewees’ perceptions of the key enabling and inhibiting factors associated with each principle. The interview included a facilitated discussion about the Collingrove definition and the National Rural Generalist Pathway: how well were these new initiatives understood; what impact did interviewees perceive on them and their communities; and what were the implications in designing and implementing a post-Fellowship support framework?

Rural practitioners on the Health Workforce Queensland database were asked to complete an online survey in order to rate each principle on a Likert scale regarding applicability in their workplace and provide further comments (see Extended data ^12^). The quantitative data from the 156 responses will be reported separately. An inductive thematic analysis approach was used to analyse qualitative data from the semi-structured interviews and online survey. The data was primarily coded by one researcher, with support and assistance provided by four other members of the project team. This provided a systematic process to generate codes from the interview data, focusing on specific characteristics related to post-Fellowship support to develop patterns of meaning by producing and revising different themes ^14^.

### Ethics

This project qualified for the exemption review pathway ‘Not Requiring Ethical Review’ (Darling Downs Health Human Research Ethics Committee, February 22, 2022: EX/2022/QTDD/81643) with collection of data not likely to adversely impact on participants’ opportunity to contribute to the project aim.

## Results

The 31 interviews conducted (12 in Town A and 19 in Town B) validated the ten interconnected guiding principles developed by the project team to underpin a post-Fellowship support framework for rural generalists. ( Table 1) ^11^. The ten themes are described below with illustrative quotes from participants.

### Principle 1: Collaboration and coordination between primary and secondary care is vital

It is critical for all sectors to work together across primary and secondary care and in the public and private sectors, particularly in rural communities. Collaborative care across the continuum provides holistic patient care and is an intended endpoint of the NRGP.

*“Primary & secondary linkage is important in remote areas and small communities, however in large rural communities, collaboration is the key, not dual appointments.” (GP_C26)*.

*“The good thing is if you want to do more hospital work, you absolutely can, where it’s possible. And the flexibility about it is very good. All doctors are working in both worlds… which I think is a very positively attractive part of our medical model, locally.” (GP_MS_M1)*. 

*“There’s been a bit of ‘us and them’ mentality between us [general practice and the hospital], which we want to move on from .. we want to really build that relationship that we have with the hospital.” (GP_B22)*.

### Principle 2: Rurally based career paths are recognised and valued

Many factors are involved in the choice of rural career. Understanding how these factors motivate and/or influence an individual is a vital component in developing attractive recruitment and retention strategies.

*“I am from a rural background…I loved my rural GP. What influenced my path to go rural, was that it is something I always wanted to do.” (GP_S29)*.

*“It's interesting work [rurally] and it's rewarding clinically. It's got a lot of variety in it ... it is enjoyable. I like the fact that it's very broad and it's everything.” (GP_VMO_M2)*.

*“Being able to do more for my patients rather than sending them to the specialist that’s what gives me the satisfaction.” (GP_M33)*.

### Principle 3: Job satisfaction is a critical element in working in rural and remote areas

Job satisfaction and the likelihood of retention are higher when employers and communities can appreciate and accommodate the personal and professional skills of rural generalists and support their aspirations. These principles are essential for any support framework.

*“Having flexible rostering/contracts allow me to work at both locations and avoid fatigue/on call when I am at the general practice plus, I have access to PD.” (SMO_S_p551)*.

*“There’s almost an expectation that doctors working in this town will do both [work in general practice and hospital]. They come and see everyone else do both. We espouse the wonders of working in both settings.” (SMO_C3)*

*“I wanted to do some obstetrics and the fact that I can sit here in a rural town and do obstetrics and emergency work and skin cancer and indigenous health and chronic disease and nursing homework and education all in one town, that’s quite rewarding for me.” (DMS_C23)*.

### Principle 4: Support is provided to attain advanced skills in order to meet community needs

While many rural doctors already demonstrate an extended scope of practice, there is currently no defined national approach to recognize and support the advanced skills of existing practitioners The evolving NRGP should provide a nationally recognised pathway for training the future workforce. National policy development is needed to understand the needs, potential barriers, and support requirements of currently practising rural practitioners who are aiming to meet the definitions laid out in the Collingrove Agreement.

*“To do secondary skills in mental health is difficult and I think that there would be a significant role for an organisation to assist with helping the step between a GP or a post-fellowship GP trying to find somewhere to do some more training.” (RMO_S13)*

*“Set a curriculum that is achievable for someone like me in private practice, and a set timeline, with an achievable goal.” (GP_VMO_M10)*.

*“Really, you need to be able to manage an airway and any acute emergency as a rural and remote doctor. …So [upskilling] at least a week in an emergency department once a year is a fair requirement.” (DMS_B14)*.

### Principle 5: The ability to attain, maintain and upgrade procedural skills in hospitals or general practice is supported to meet community needs

Rural doctors utilise more procedural skills than their urban colleagues. Identifying, developing, and maintaining procedural skills is needed to ensure doctors remain safe and competent. While posing a challenge, this approach can enable development of appropriate solutions to deliver upskilling locally as an integral part of a support framework.

*“The bit I probably find the most overwhelming at the moment is the logistic planning about trying to do some upskilling.” (GP_VMO_G7)*.

*“One of the biggest problems that doctors have is getting away to do is a clinical attachment, and I think the [Coordination Unit] could definitely support clinical attachments.” (DMS_C23)*.

*“We run PROMPT [obstetric course] right here in town … so that’s great we get to do that stuff here locally.” (GP_VMO_G7)*.

### Principle 6: The ability to work across hospital and general practice facilities to provide coordinated care is supported

The Collingrove statement requires a rural generalist to deliver comprehensive general practice and emergency medicine service as well as additional specialty care in both community (primary care) and hospital (secondary care) settings. A support framework must therefore address relevant industrial and employment elements to enable rural generalists to work collaboratively across all sectors.

*“Offer support and training for staff to run GP practice in a rural setting to make them viable and have a reliable staff that can contribute to ease the workload. Incentives or support for allied health workers to support GP in the community (GP_H15)*

*“Developing sustainable workforce options to improve private GP options for patients would improve their wellbeing and care. Medicare bulk-billing practices are not sustainable to adequately compensate professionals wanting to work as a private GP.” (GP_S_p558)*.

*“Let’s face it, private practice is private practice, and it’s a business, with accounting, insurances, lease agreements and a lot of terrifying stuff in there. We need to be able to mentor someone through it, so they don’t have a nervous breakdown.” (MS_F24)*.

### Principle 7: Education and training needs are supported

An appropriate approach to training and education needs to be developed in order to provide the range of services a rural community requires. An effective support framework must enable rural generalists to source, develop and maintain the skills required to provide medical services relevant to their community’s evolving needs.

*“A structured mentoring program for all clinicians, wherever they sit in the hierarchy. It would pay enormous dividends.” (DMS_B14)*.

*“With respect to my other experienced rural GP colleagues, we back each other up very well. But that’s not a new thing, country doctors have always done that.” (GPVMO_M2)*

### Principle 8: Professional peer support is available

The demands and professionally isolation of rural medicine have been well documented, as have the rewards and satisfaction. An effective support framework needs to facilitate connection of rural generalists with professional peer supports and key organizations.

*“I think it is that collegial support that helps... somebody that you can trust, and that you can raise concerns with, and that you can discuss patients with.” (GP_VMO_N11)*.

*”I have met some really fantastic people. And you’d look at their practice and think, that is really good practice and I take on board what they were doing.” (GP_VMO_M10)*.

*“I love this place because of the team, despite the fact that we are stressed, we do have a bond of some sort. Even [during] the challenge of the current times, I guess that we have bonded even more.” (EDMS_S31)*.

### Principle 9: The viability of existing rural general practice is enhanced

Thriving private practices are critical to meeting the needs of rural communities. Rural GPs must co-ordinate their patients’ care while working collaboratively with the public hospital workforce. Developing sustainable approaches and cultural change which provide support for general practices to remain viable is critical for ensuring quality primary care. An effective post-Fellowship support framework should minimise barriers and maximise enablers of private practice viability.

*“The current remuneration model makes general practice unappealing to doctors of my generation. When working for the public health system I turn up, work hard, get paid well and supported with leave. The comparison with general practice is stark.” (SMO_S_p549)*.

*“Rural generalism should not only be recognised as an equivalent specialist, it actually should be probably the most top-paying speciality, because it’s the most difficult speciality.” [Anonymous]*

*“Doctors working in private practice should be supported financially just as much as doctors who work at least partly for {HHS]. rural generalists employed in the hospital receive more benefits and it seems much easier for doctors in the [HHS] system to obtain support.” (GP_VMO_M10)*.

### Principle 10: Clinical leadership capability is recognised, enhanced, encouraged, and supported

The importance of effective local leadership to enable a positive organisational culture cannot be overstated. Skilled leaders who encourage innovation, quality, and productivity to flourish in an organization, create a culture in which staff are likely to feel motivated, empowered, and supported ^15^. This has the effect of improving attraction and retention of a skilled workforce ^16^ and promotes quality and safety of patient care ^17^.

*“Clinical leadership is vitally important to attract and retain people. If they don’t feel there’s good leadership, or feel they’re not supported, that’s reason they leave.” (DMS_C23)*.

*“Important to have good clinical leadership in our town because it makes you feel supported. If you don’t have good clinical leadership, you don’t have happy doctors. And if you don’t work with happy doctors your juniors don’t want to come back.“ (RMO_S13)*.

### Themes

Four main themes emerged from inductive thematic analysis of the qualitative data:

1. Connecting Primary and Secondary Care
2. Valuing a Rural Career
3. Supporting Training and Education
4. Valuing Rural General Practice.

Each of these overarching themes can be mapped to one or more of the Guiding Principles. Multiple subthemes also emerged, key factors impacting on and contributing to the associated themes ( Table 3).

**Table 3.** Themes and subthemes.

Theme 1: Connecting Primary and Secondary Care	
*Guiding Principles*	*Subthemes*
Principle 1: Collaboration and coordination between primary and secondary care is vital Principle 6: The ability to work across hospital and general practice facilities to provide coordinated care is supported Principle 10: Clinical leadership capability is recognised, enhanced, encouraged, and supported	Collaborative Models and Systems ▪ System integrations and shared information ▪ The public-private interface Trust, Leadership and Culture ▪ Welcoming doctors to work across care settings ▪ Facilitates doctors working across care settings ▪ Streamlining the credentialing process ▪ Employment strategies
Theme 2: Valuing a Rural Career	
*Guiding Principles*	*Subthemes*
Principle 2: Rurally based career paths are valued Principle 3: Job satisfaction is a critical element in working in rural and remote areas	▪ Personal Factors · Lifestyle and community · Family considerations ▪ Professional Factors · Rural practice experience · Practice variety · Work conditions · Collegial support
Theme 3: Supporting Training and Education in a Rural Area		
*Guiding Principles*	*Subthemes*
Principle 4: Support is provided to attain advanced skills in order to meet community needs Principle 5: The ability to attain, maintain and upgrade procedural skills is supported to meet community needs Principle 7: Education and training needs are supported Principle 8: Professional peer support is respected, valued and available	▪ Training challenges unique to rural medicine · Cost · Training option flexibility · Management support ▪ Building capacity for rural doctor training · Locally available training · Mentoring · Coordination Unit function
Theme 4: Valuing Rural General Practice	
*Guiding Principles*	*Subthemes*
Principle 9: The viability of existing rural general practice is enhanced	▪ Rural GP in Practice · Recognition and reward · Attraction and workforce supply ▪ General Practice Viability · Funding models · Business support

### Theme 1: Connecting primary and secondary care

Rural generalists need to work across primary and secondary care, with integration facilitated by cross-collaboration, streamlined credentialing processes and flexible employment models underpinned by effective medical leadership with strong trust and culture.

*“The more fractional appointments you have, the more flexibility you have for running an acute service, but also giving an opportunity for the fractional appointments to work in primary care. And so that particular model, I think, works very well.” (GP_B22).*

*“The IT systems are integrated between the general practice and the hospital, so we can access patient notes between the two, which just makes that transition of care pretty seamless really, which I think is a big advantage.” (GP_C26).*

*“Hospital Health Services need to work out how to do a blended model, so private practitioners can come back into their facilities and look after their own patients. And so, I think the public/private divide is destructive.” (GP_VMO_M10).*

### Theme 2: Valuing a rural career

Rural doctors see their work as exciting, challenging, stimulating, and intellectually demanding and were very satisfied with opportunities to use their skills. However, they were concerned about access to schooling and employment opportunities for their partner. Personal factors and work-life balance are essential for job satisfaction and retention.

*“You don't want kudos or thanks, because that’s part of your job, but there’s a good feeling that you're contributing to the community.” (ED_F28).*

*“I think for me it was the lifestyle, it was the job satisfaction, it was never about the money.” (DMS_C23).*

*“The job that I have here is perfect, it’s exactly as it was advertised and that’s pretty much it.” (GP_SMO_H5)*

### Theme 3: Supporting training and education

Rural training needs to be bespoke, recognise time and financial pressures, flexible and supported. Capacity is increased where training is locally available and family-friendly with support in navigating training pathways. Coordinating information about sites and strategies, with innovative and flexible approaches and supported professional development opportunities are essential.

*“A structured mentoring program for all clinicians, wherever they sit in the hierarchy. It would pay enormous dividends.” (DMS_B14).*

*“I’m missing having an actual designated mentor. That would be helpful for personal, professional and academic reasons. Someone who is five to ten years further along.” (GPSMO_H5).*

*“I'd love to be able to just have time with experienced senior doctors, who not just offer advice for specific cases but also to talk to you about self-reflection.” (GP_S29).*

### Theme 4: Valuing rural general practice

Not all rural doctors wish to work both privately and publicly. Many cite no interest in hospital emergency care unless skilled and trained. Fulfilling Collingrove requirements may enable workforce solutions that increase the status of rural generalist medicine and facilitate transferability of Rural Generalists across jurisdictions. Workforce challenges include attracting, recognizing, and rewarding rural GPs to ensure workforce supply and developing funding models and business supports that support practice viability.

*“Valued by your community for being there. Yeah, the value of the people and your own internal feeling of the value of the work that you actually do, we’re the true generalists.” (GP_SMO_H5)*

*“Rural generalism should not only be recognised as an equivalent specialist, it actually should be probably the most top-paying speciality, because it’s the most difficult speciality.” [Anonymous]*

*“We're the true generalists … aren’t we? We are actually skilled in a lot of areas.” (VMO_GP_M2).*

## Discussion

The guiding principles developed by the project team and validated by the interviewees enabled development of a grounded, contextually relevant approach to post-Fellowship support framework for rural generalists working in other jurisdictions.

The principles were designed in the real-world context of a mature Queensland Rural Generalist Pathway and an evolving Australian National pathway, so are highly relevant to these jurisdictions and validated with relevant Queensland stakeholders.

These principles are inter-connected as indicated in the four overarching themes - Connecting Primary and Secondary Care; Valuing a Rural Career; Supporting Training and Education; and Valuing Rural General Practice - and would be incomplete when considered in isolation. The principles, whilst designed in the real-world context of a mature QRGP and evolving NRGP, encourage an ongoing conversation about the challenges and priorities identified by rural doctors. Engagement and consultation with rural stakeholders is needed as a next step to discuss and achieve consensus on the Coordination Unit support functions and governance.

This paper highlights the importance of retention as a workforce strategy, underpinned by the guiding principles and themes that have been identified. These principles and themes illustrate some important new insights into workforce models and the role of coordination units. Specific strategies around skill acquisition and practice models are described, as is the importance of offering flexible pathways to acquire the necessary advanced skills.

While integrated training concepts are important, policy makers, health administrators and funders need to go further and ensure rural communities can access integrated and sustainable medical care via joined-up practice models. This will require future commitment and investment in innovative service and workforce design that includes support elements to enhance and retain Rural Generalists.

The implementation of the NRGP provides an opportunity to expand the coordination function beyond vocational training and support, as is currently the case in Queensland. The next phase in a national roll-out could well involve coordination of rural generalist workforce models in order to develop and support approaches that will attract, retain, and sustain rural generalists capable of meeting community needs.

The four themes identified reflect the key areas of support which rural doctors felt were necessary to support post-Fellowship doctors to practise in accordance with the Collingrove Agreement. These themes are the foundations upon which the post-Fellowship support framework will be built with the aim of facilitating two specific retention strategies:

- Supporting existing rural doctors in both primary and secondary care to attain the necessary skills and develop practice models aligned to the Collingrove Rural Generalist definition. This would enable national recognition as a Rural Generalist for those rural doctors who wished to do so.
- Establish a coordinated national approach that delivers a highly trained integrated medical workforce likely to remain in rural practice beyond Fellowship and who are capable of adjusting to emerging community changes, expectations, and evolving needs.

Two further quotes from participants illustrate the importance of this coordination function:

*“It is very hard to be a hospital procedural doctor in Queensland and work in General Practice; Mandatory training and Queensland Health credentialing requirements are almost prohibitively demanding for one person with two roles.” (SMO_p557).*

*“it’s a bit strange that you can get approved [credentialled] for one health service and not in another [health service]. Coordination between the credentialing committees is a problem.” (GP_MS_M1).*

### Importance of retention

These findings mirrored those from other studies that doctors currently working in rural communities intend to remain in those sites for several more years. This in turn strongly argues for further focused retention strategies to support the long-term commitment of rural doctors in both private and public sectors ^18^.

Such approaches support Australia’s well-established recruitment and training strategies. Rural Generalists also need to be able to acquire and maintain advanced skills at any stage in their career in order to develop a resilient and self-sustaining multi-skilled rural workforce able to meet local service gaps and address the broader challenges of rural generalist practice ^18^.

One important training focus should be on exploring opportunities to overcome challenges of completing advanced skill / procedural training in terms of the time constraints and the need for rural doctors to travel to larger sites. Strategies are needed which remove inequality in terms of access to or cost of continuing professional development activities, which will require cooperation between colleges, training institutions and jurisdictions.

This project’s findings indicate that both access to flexible training pathways and flexible funding solutions would be required for private rural GPs to practise in accordance with the Collingrove Agreement and meet the NRGP endpoint.

The National Rural Generalist Pathway can lead the development of a stronger and better-distributed rural medical workforce, as increasing the numbers of Rural Generalists would ensure more sustainable workloads and improve retention ^5^. However, rural doctors should be aware of national developments like the Collingrove agreement and the NRGP for this to occur, and they further need access to the support required to navigate the pathway requirements.

The next phase of this project will progress the design and development of the post- Fellowship support framework and describe the quantitative survey findings.

## Conclusion

The widespread international rural medical workforce shortage has consequent effects on service delivery in both primary and secondary care. Recruitment and retention strategies form the cornerstone of addressing these workforce shortages. Increasing recognition of the differences between recruitment and retention has led to a growing appreciation of the different approaches needed for each.

This report presents ten guiding principles for the development of a support framework for rural generalists who have achieved Fellowship. The principles encourage an ongoing conversation about the priorities and challenges raised by rural doctors.

These real-world principles undertaken by two rural workforce organisations in Queensland, drawn from the local experience in the context of an evolving national pathway may well be relevant in other jurisdictions and would be worth exploring in other settings.

This Post Fellowship Support Framework was designed to support and sustain practice integration for rural doctors across primary and secondary service domains in their post graduate careers. The framework addresses domains that focus on procedural practice and advanced skills enabling doctors to provide comprehensive general practice, specialised care including emergency care in hospital and community settings. This project utilised a mixed methods approach to validate these guiding principles.

Considerable effort is already directed towards recruiting and training rural doctors. These principles may aid retention, ensuring rural doctors are, in the word of pioneering Australian rural doctor Col Owen, ‘comfortable and contented,’ as well as ‘confident and competent’ [Dr Col Owen, *pers comm* 2022].

### Limitations

This study was only in one Australian jurisdiction with data derived from interviews with medical staff. Future work could explore other perspectives such as that of the funders, other health professionals and the community. While there were efforts to sample widely across Queensland rural doctors, it is not clear how representative the survey population was. In addition, some of the 31 doctors interviewed had limited knowledge and variable interest in meeting the NRGP requirements of the Pathway. Others stated that recruiting a hospital workforce that meets community service needs or concentrating on private practice sustainability were areas of higher priority.

## Data Availability

The underlying data for this article is from the *Post Fellowship Support Framework for Rural Doctors. A Queensland Pilot Project,* February 2021. Zenodo: A Post-Fellowship Support Framework for Rural Doctors: the Queensland experience. Appendices https://doi.org/10.5281/zenodo.14053515 ^12^ This project contains the following extended data: The interview questions used in developing and validating the Post Fellowship Support Framework The online survey questions used in developing and validating the Post Fellowship Support Framework COREQ (COnsolidated criteria for REporting Qualitative research) checklist Data are available under the terms of the Creative Commons Attribution 4.0 International license (CC-BY 4.0).
