# Supplementary material for: A Post-Fellowship Support Framework for Rural Doctors: the Queensland experience
Source: MedEdPublish (2016). 2024 Nov 20;14:6. Originally published 2024 Feb 19. [Version 2] doi: 10.12688/mep.20025.2 (PMC11099509; doi:10.12688/mep.20025.2)
Supplement: Peer review history

# Reviewer report: Reviewer response for version 2

**DOI:** 10.21956/mep.22267.r40181

**Published:** 2025-01-10

**Authors:** Andrew Kirke

**Affiliations:** 1 The Rural Clinical School of WA, The University of Western Australia, Perth, Western Australia, Australia

Thank you for the opportunity to review the edited version of this manuscript. The edited manuscript has addressed my concerns with version one, notably

1. The qualitative evidence has been included, specifically quotations from participants.
2. The manuscript is now a stand-alone document and as such is easier to read and refer to.
3. Minor inconsistencies noted between version 1 and the supporting evidence in a previous report have been corrected.
4. The use of the Town A and Town B methodology has been explained and justified.

I have no further concerns and am happy for version two to be presented as the published version.

Have any limitations of the research been acknowledged?

Yes

Is the work clearly and accurately presented and does it cite the current literature?

Yes

If applicable, is the statistical analysis and its interpretation appropriate?

Not applicable

Are all the source data underlying the results available to ensure full reproducibility?

Partly

Is the study design appropriate and is the work technically sound?

Partly

Are the conclusions drawn adequately supported by the results?

Partly

Are sufficient details of methods and analysis provided to allow replication by others?

Partly

Reviewer Expertise:

Rural health, rural obstetrics, rural workforce, screening in primary health care

I confirm that I have read this submission and believe that I have an appropriate level of expertise to confirm that it is of an acceptable scientific standard.

# Reviewer report: Reviewer response for version 2

**DOI:** 10.21956/mep.22267.r40183

**Published:** 2024-12-02

**Authors:** Garry Nixon

**Affiliations:** 1 Department of General Practice and Rural Health, University of Otago, Dunedin, New Zealand

Thank you for undertaking this revision. The addition of quotes in particular, has considerably improved the paper.

Have any limitations of the research been acknowledged?

Yes

Is the work clearly and accurately presented and does it cite the current literature?

Partly

If applicable, is the statistical analysis and its interpretation appropriate?

Not applicable

Are all the source data underlying the results available to ensure full reproducibility?

Yes

Is the study design appropriate and is the work technically sound?

Partly

Are the conclusions drawn adequately supported by the results?

Partly

Are sufficient details of methods and analysis provided to allow replication by others?

No

Reviewer Expertise:

Rural health research.

I confirm that I have read this submission and believe that I have an appropriate level of expertise to confirm that it is of an acceptable scientific standard.

# Reviewer report: Reviewer response for version 2

**DOI:** 10.21956/mep.22267.r40212

**Published:** 2024-12-02

**Authors:** Cheri Bethune

**Affiliations:** 1 Memorial University, Newfoundland and Labrador, Canada

The authors have responded appropriately to previous reviewer's comments to my satisfaction.

Have any limitations of the research been acknowledged?

Yes

Is the work clearly and accurately presented and does it cite the current literature?

Yes

If applicable, is the statistical analysis and its interpretation appropriate?

Not applicable

Are all the source data underlying the results available to ensure full reproducibility?

Yes

Is the study design appropriate and is the work technically sound?

Yes

Are the conclusions drawn adequately supported by the results?

Yes

Are sufficient details of methods and analysis provided to allow replication by others?

Yes

Reviewer Expertise:

NA

I confirm that I have read this submission and believe that I have an appropriate level of expertise to confirm that it is of an acceptable scientific standard.

# Reviewer report: Reviewer response for version 2

**DOI:** 10.21956/mep.22267.r40213

**Published:** 2024-11-30

**Authors:** Jessica Beattie

**Affiliations:** 1 Deakin University, Burwood, Victoria, Australia

I agree with the authors’ rationale that retention strategies in rural healthcare have received insufficient attention, and that further empirical research in this area is essential.

 As such, the paper’s focus on retention strategies is highly relevant and contributes meaningfully to the field but there are some aspects of the paper the require attention.

**Abstract:**

**Results: **themes do not merely "emerge" but are co-constructed through the interaction between participants and researchers.

**Introduction:**

 The introduction provides a solid foundation for the study, presenting clear evidence and rationale for the research.

**Methods:**

 If the journal’s formatting guidelines permit, I suggest breaking the Methods section into additional subheadings, such as "Data Collection" and "Data Analysis," to improve clarity and structure.

 Additionally, the authors abbreviate the National Rural Generalist Pathway (NRGP) in the introduction but later refer to it inconsistently as either "the national pathway" or by its full title. Consistency in terminology would improve readability.

 The concept of "ascribing weighting to the principals" is introduced within a section discussing qualitative components, but the term "weighting" typically suggests a quantitative approach. Later, the authors mention using a Likert scale, which will be reported separately. It would be helpful to clarify whether this Likert scale refers to the same aspect of the study, and to ensure that the quantitative elements are not mixed throughout the qualitative methods section. It may be beneficial to mention the quantitative component in a single, clear statement without introducing it multiple times.

 Under the "Approach" section, the authors refer to "data saturation," a term that has been criticised in qualitative research literature. While the authors used the COREQ guidelines (as a previous reviewer suggested), I recommend including a reference supporting the use of data saturation. Many researchers ascribe to "theme saturation".

 More detail is needed regarding the data analysis process, particularly since it is described as "inductive." Further elaboration on how the analysis was conducted would enhance the transparency and rigor of the methodology. From looking at the interview guide I didn’t really understand how the principles were qualitatively validated.

**Themes:**

 As noted earlier, themes are co-constructed between participants and researchers, rather than emerging naturally from the data. Inclusion of a reflexive statement from the authors would be helpful.

 Since the themes align with predefined principles, it is important to clarify whether the thematic development was truly inductive or if it followed a more deductive approach. I suggest the authors justify this choice by referencing appropriate literature.

 Regarding Theme 4, the quote, "Rural generalism should not only be recognised as an equivalent specialist, but it actually should also probably be the most top paying speciality, because it’s the most difficult speciality" [Anonymous], has already been used earlier in the paper under the "viability of existing rural practice" theme. I recommend removing this quote or replacing it with a new one. Furthermore, the description of Theme 4 reads more like a general statement and does not clearly align with the participants’ voices. It would be helpful to expand on the meaning of the term "true generalist" as used by the participants, as this concept is not fully explored.

 The authors assert that the participants validated the principles; however, the results section does not clearly reflect this. For example, the language used in the results section does not seem to capture the participants' voices as expected. It would have imagined the description would have read a bit more like "Participants agreed that X was a guiding principle due to…"

**Discussion:**

 Discussion could be strengthened by a more detailed interpretation of the results in relation to the broader literature. Currently, some sections are somewhat repetitive, and paragraphs 2 and 3, in particular, could be consolidated.

 The two bullet points in the Discussion section warrant further elaboration.

- In the first point, the authors discuss the potential for national recognition of rural generalists. This is an important issue and should be expanded upon to explore how the recognition process might unfold and what the criteria for such recognition might be. It is my understanding a proposal has already been put to the Australian government to have RG recognised.
- In the second point, I recommend that the authors discuss the barriers to achieving a coordinated approach to rural generalism, drawing on relevant literature and then remove the participant quotes.

Have any limitations of the research been acknowledged?

Yes

Is the work clearly and accurately presented and does it cite the current literature?

Partly

If applicable, is the statistical analysis and its interpretation appropriate?

Not applicable

Are all the source data underlying the results available to ensure full reproducibility?

No source data required

Is the study design appropriate and is the work technically sound?

Partly

Are the conclusions drawn adequately supported by the results?

Yes

Are sufficient details of methods and analysis provided to allow replication by others?

Partly

Reviewer Expertise:

Qualitative rural medical education research

I confirm that I have read this submission and believe that I have an appropriate level of expertise to confirm that it is of an acceptable scientific standard, however I have significant reservations, as outlined above.

# Reviewer report: Reviewer response for version 2

**DOI:** 10.21956/mep.22267.r40182

**Published:** 2024-11-21

**Authors:** James Rourke

**Affiliations:** 1 Faculty of Medicine, Memorial University Newfoundland, St. John's, Newfoundland and Labrador, Canada

The added direct quotes will resonate with rural doctors in Queensland and far beyond and inform rural health educators and system administrators.

Have any limitations of the research been acknowledged?

Yes

Is the work clearly and accurately presented and does it cite the current literature?

Yes

If applicable, is the statistical analysis and its interpretation appropriate?

Yes

Are all the source data underlying the results available to ensure full reproducibility?

Yes

Is the study design appropriate and is the work technically sound?

Yes

Are the conclusions drawn adequately supported by the results?

Yes

Are sufficient details of methods and analysis provided to allow replication by others?

Yes

Reviewer Expertise:

Rural Medicine, Medical Education, Social Accountalbilty,

I confirm that I have read this submission and believe that I have an appropriate level of expertise to confirm that it is of an acceptable scientific standard.

# Reviewer report: Reviewer response for version 1

**DOI:** 10.21956/mep.21442.r36195

**Published:** 2024-05-16

**Authors:** Garry Nixon

**Affiliations:** 1 Department of General Practice and Rural Health, University of Otago, Dunedin, New Zealand

As the authors note retention of the rural medical workforce is an important and understudied area of rural health research. This paper is therefore an important contribution.

Overall:

 I suggest the authors review their paper in line with the COREQ checklist and include a completed copy of the checklist with any future submission. All sections would benefit from this exercise but the methods and results sections in particular.

Abstract:

 Methods – should outline methods relating to the research presented in this paper  (not so much the larger one it is part of).

 Results -  should include the main findings of the study. These appear more in the conclusion.

Introduction:

 Provides excellent background on the Australian context and Rural Generalist Programme, as well as literature or recruitment and retention.

Methods

 Is the Delphi project a part of the study being reported here? If so the methods and the results need to be reported in the appropriate section. That is table 1 should be in results and the Delphi method described in the methods. But I get the impression it is not and is work done prior to this study – in which case it needs to be referenced.

 The study aim should be at the end of the introduction. In fact much of the early methods section would be better placed in the introduction.

 I appreciate that this is part of a larger study that may be mixed methods but the research presented here is a qualitative interview based study?  

Results:

 I am unclear if the qualitative parts of the survey formed part of this study or not?  If the survey is included then its results need to be presented in the results section and care taken to explain how the interview and survey results were integrated.

 A major omission from the results is the data – in a qualitative interview based study the data is normally presented as quotations. It is not possible to determine if the data and the findings are consistent.

Discussion:

 The discussion could be improved by starting with a brief summary of the main findings (table 3 might be better in the results section), consideration of what new knowledge the study adds in relation to existing literature and then moving to policy implications.

Have any limitations of the research been acknowledged?

Yes

Is the work clearly and accurately presented and does it cite the current literature?

Partly

If applicable, is the statistical analysis and its interpretation appropriate?

Not applicable

Are all the source data underlying the results available to ensure full reproducibility?

Yes

Is the study design appropriate and is the work technically sound?

Partly

Are the conclusions drawn adequately supported by the results?

Partly

Are sufficient details of methods and analysis provided to allow replication by others?

No

Reviewer Expertise:

Rural health research.

I confirm that I have read this submission and believe that I have an appropriate level of expertise to confirm that it is of an acceptable scientific standard, however I have significant reservations, as outlined above.

# Response

**Published:** 2024-11-11

**Authors:** Tarun Sengupta

**Affiliations:** James Cook University, Townsville City, Queensland, Australia

**Response:**

- We thank Prof Nixon for his thoughtful review and acknowledgement of the importance of addressing retention as a workforce strategy.
- We have incorporated his suggestions into the revised paper as follows:   We have added over thirty quotations illustrating the relevant principles and themes throughout the paper as recommended.
- We have also completed a COREQ (COnsolidated criteria for REporting Qualitative research) checklist which appears in the extended data ( https://doi.org/10.5281/zenodo14053515).
- A number of minor amendments were made to the text to ensure consistency with the checklist.  
- There is some reordering of the text and headings, and additional clarifications as recommended in the Abstract, Methods, and Discussion sections.
- We have added a line to the abstract noting that this study focuses on the qualitative components, which is also mentioned in the Methods section and the last paragraph of the Discussion.
- Interestingly Table 3 does appear in the Results section in the online version of this paper as suggested (but note that printed versions may be different).

# Reviewer report: Reviewer response for version 1

**DOI:** 10.21956/mep.21442.r36196

**Published:** 2024-05-01

**Authors:** James Rourke

**Affiliations:** 1 Faculty of Medicine, Memorial University Newfoundland, St. John's, Newfoundland and Labrador, Canada

This article addresses the issue of rural doctor retention with a framework for developing a post-fellowship support program. Ten guiding principles were developed in a pilot project completed in 2021 in Queensland, Australia. The article provides a summary of the qualitative component of that project. 31 rural doctors in various locations were interviewed and validated the guiding principles. A thematic analysis produced four main themes. As noted, this study was limited to one Australian state. However, the guidelines and themes are relevant to rural practice and will interest readers worldwide. A link is provided to the full pilot project report as the underlying data, which provides expanded interview input, including informative quotes from the rural doctors. The article could be made more interesting by including some of these quotes.

Have any limitations of the research been acknowledged?

Yes

Is the work clearly and accurately presented and does it cite the current literature?

Yes

If applicable, is the statistical analysis and its interpretation appropriate?

Yes

Are all the source data underlying the results available to ensure full reproducibility?

Yes

Is the study design appropriate and is the work technically sound?

Yes

Are the conclusions drawn adequately supported by the results?

Yes

Are sufficient details of methods and analysis provided to allow replication by others?

Yes

Reviewer Expertise:

Rural Medicine, Medical Education, Social Accountalbilty,

I confirm that I have read this submission and believe that I have an appropriate level of expertise to confirm that it is of an acceptable scientific standard.

# Response

**Published:** 2024-05-10

**Authors:** Tarun Sengupta

**Affiliations:** James Cook University, Townsville City, Queensland, Australia

We thank Professor Rourke for his kind review, and for highlighting the importance of retention of rural doctors world-wide. We note the comments on adding some additional quotes to illuminate the material and will incorporate this in the next version of the paper.

# Reviewer report: Reviewer response for version 1

**DOI:** 10.21956/mep.21442.r36200

**Published:** 2024-03-12

**Authors:** Andrew Kirke

**Affiliations:** 1 The Rural Clinical School of WA, The University of Western Australia, Perth, Western Australia, Australia

I note that this manuscript is a subset of a larger previously published Health Dept report "Post Fellowship Support Framework for Rural Doctors", cited as the evidence source for this paper. The rationale for the study is well articulated. The methodology is mostly clear and the conclusions seem appropriate. However I have questions on the following points:

 1. As a stand alone paper the evidence such as example quotes supporting themes and analysis are absent. They are present in the original report but absent here. Is it expected that the reader is supposed to refer back to the original report?

 2. In the methodology there are some minor inconsistencies between the original Health Dept report and this paper, example below.

 Report "A group of 32 doctors (17 male and 15 female) from Towns A and B were interviewed"

 Manuscript "A total of 31 doctors (15 female and 16 male) from Towns A and B were interviewed."

 3. In neither the source data of the original report or the manuscript here, is the rationale for dividing the towns interviewed into Town A, a real town, and Town B, a virtual town comprised of data from 6 towns, clearly explained. How it was done is explained but not why. It is not clear how this is relevant to the qualitative analysis presented here.

 I think this study has merit and should be reported in the peer reviewed literature however as a stand alone paper the current iteration feels incomplete. If this is a stand alone qualitative study then evidence of the original data should be provided and minor inconsistencies between the report and the paper resolved.

Have any limitations of the research been acknowledged?

Yes

Is the work clearly and accurately presented and does it cite the current literature?

Yes

If applicable, is the statistical analysis and its interpretation appropriate?

Not applicable

Are all the source data underlying the results available to ensure full reproducibility?

Partly

Is the study design appropriate and is the work technically sound?

Partly

Are the conclusions drawn adequately supported by the results?

Partly

Are sufficient details of methods and analysis provided to allow replication by others?

Partly

Reviewer Expertise:

Rural health, rural obstetrics, rural workforce, screening in primary health care

I confirm that I have read this submission and believe that I have an appropriate level of expertise to confirm that it is of an acceptable scientific standard, however I have significant reservations, as outlined above.

# Response

**Published:** 2024-04-12

**Authors:** Tarun Sengupta

**Affiliations:** James Cook University, Townsville City, Queensland, Australia

We thank the reviewer for these constructive comments and for taking the time to review this paper as well as the source document. We agree that including evidence such as example quotes supporting themes and analysis as presented in the original report could strengthen this paper. We were trying to strike a balance between information and word limits and were already concerned about the length of the paper. The original report, readily available online, was over 30 000 words, but we will consider this point in future versions of this paper.  The number of participants (31) in our manuscript is correct. While we initially had confirmation from 32 doctors, one of them withdrew and unfortunately, this change was not updated in the report, leading to an error in the recorded number of doctors interviewed. We thank the reviewer for noting this and will correct the online report. The choice of Town A and Town B reflected a ’purposive sampling strategy’ to get the best representation of communities across Qld, in order to ensure the study encompassed both high-functioning communities and other types of communities. This was a deliberate  strategy with the primary objective to obtain a comprehensive representation of communities across the state. By including towns from various geographic areas, the study aimed to better capture the diversity of medical models implemented in different locations and gain insights into engagement models for doctors across a wide spectrum of rural Queensland.
